# Effects of exergames on student physical education learning in the context of the artificial intelligence era: a meta-analysis

**DOI:** 10.1038/s41598-024-57357-8

**Published:** 2024-03-26

**Authors:** Mengnan Zhao, Xurui Lu, Qi Zhang, Rutong Zhao, Bohang Wu, Sheng Huang, Sunnan Li

**Affiliations:** 1 College of P.E. and Sports, Beijing Normal University, Beijing, 100875 China; 2 College of P.E., Yanshan University, Qinhuangdao, 066000 China; 3 Tianjin Experiment High School, Tianjin, 300074 China; 4 Shenzhen Senior High School Group, Shenzhen, 518040 China; 5 Chongqing Nankai Middle School, Chongqing, 400065 China

**Keywords:** Human behaviour, Quality of life

## Abstract

Whether the application of exergames in physical education (PE) courses can significantly improve student performance in PE learning is still controversial. This review explores the promoting effect of exergames on student PE learning and the conditions in which the effect of exergames can be maximized. Based on the PICOS method, two researchers independently searched the ProQuest database, EBSCO database, Web of Science (WoS) database, PubMed database, Chinese National Knowledge Infrastructure (CNKI) database, Wanfang database, and VIP database, evaluated the literature quality using the Cochrane system evaluation manual, and performed a meta-analysis of the included literature. A total of 16 randomized controlled trials involving 2962 subjects were included in this study. The meta-analysis showed that exergames effectively improved student performance in PE learning (SMD = 0.45, 95% CI: 0.27–0.63, P < 0.00001). Subgroup analysis indicated that better results could be achieved when exergames were introduced in small kindergarten classes and continued for 1–2 months.

## Introduction

According to the 2023 World Obesity Federation research report, more than 340 million children and adolescents aged 5–19 are currently overweight or obese, and this trend continues to rise^1^. The World Health Organization (WHO) suggested that insufficient physical activity is a major contributor to this trend^2^. They recommended that children engage in at least 60 min of moderate physical activity per day to maintain a healthy weight^3^. However, data showed that 81% of adolescents do not meet the WHO recommended amount of daily physical activity^4^. Therefore, it is urgent to promote physical activity among children and adolescents.

With the rapid development of big data, the Internet of Things, artificial intelligence, and virtual reality, introducing new technologies into teaching has become a new development trend^5^. Exergames is an emerging product of the information age. It combines exercise and gaming and can also be called active video gaming^6^. Currently, the academic community defines exergames as electronic games that encourage players to improve non-sedentary physical activities, such as strength, balance, and flexibility^7^. It is a new type of video game that allows the body to control games and requires the participation of large muscle movements. Unlike sedentary electronic games that contribute to obesity in children and adolescents, exergames create more realistic and diverse scenarios and require practitioners to participate in virtual sports, fitness exercises, and other interactive activities. As the enjoyment and persistence of the user increase and the boredom of repetitive body movements decreases, the users can have frequent physical activities, thus achieving sufficient energy consumption levels^8^. Video gaming technology has advanced from motion-sensing handles to optical sensors with the development of technology, allowing for more realistic whole-body movement. This development has prompted researchers to explore the potential applications of exergames and exergames in physical education (PE, the following "physical education" will be denoted by "PE".)^9^. In school PE, there are still difficulties, such as tedious PE courses, failure to effectively stimulate student "interest" in sports, and lack of sports experience among students in PE courses. Therefore, exergames can be a solution for problems in school PE classes^10^, as well as a positive strategy and potential tool to promote student learning in PE^11^.

Previously, scholars have explored the application of exergames in clinical rehabilitation^12^, health promotion^13^, and cognitive training^14^. The results showed that exergames increased physical activity, energy expenditure, maximum oxygen uptake, and heart rate in users^15,16^. However, empirical evidence supporting its effectiveness is limited^17^, and its application in education is still an emerging approach^18^. For some students, the impact of replacing traditional physical education teaching with exergames may lead to some concerns. Although there is considerable evidence suggesting that exergames can enhance the mental and physical health of children, some studies also argued that exergames could not fully improve physical activity levels to achieve significant health benefits. On this basis, whether exergames are applicable and useful for school PE remains unclear^19,20^. In addition, academic research on specific issues such as "what class size is suitable" and "what stage of study is suitable" for exergames is still lacking. In order to effectively conduct relevant research and practice, the practical utility of exergames on student PE learning still needs to be revealed. In this study, the impact of exergames on student PE learning outcomes was investigated through meta-analysis, further clarifying the role of exergames in different stages, class sizes, and experimental cycles. The findings of this study can provide a reference for the application and further development of exergames in the current PE teaching process.

## Methods

### Data sources

The databases include Chinese National Knowledge Infrastructure (CNKI), Wanfang, VIP, Web of Science (WoS), ProQuest, PubMed, and EBSCO, and the search period was set from January 2001 to November 2023. To ensure the comprehensiveness of the literature search, the subject terms of the Chinese search were set as "games", containing "sports", "effect or performance or application or practice or impact", etc. The search for foreign articles was mainly conducted with subject headings 1 (game or gamification or game-based or gamifying or gamified or exergame or exergaming or AVG), subject headings 2 ("physical education" or PE), and subject headings 3 (performance or outcome or achievement or effect or score or experiment). The search also covered relevant reviews and references of included studies to minimize the number of missed articles. The languages of all included literature were Chinese and English. To ensure the high quality and scientific validity of the included studies, the included Chinese literature was obtained from core journals and CSSCI journals, and the English literature was sourced from SCI and SSCI journals.

### Inclusion and exclusion criteria

The inclusion and exclusion criteria were determined according to the research topic, i.e., the specific indicators of the research topic were determined based on the inclusion criteria, as well as the exclusion of factors in the research topic that influenced the results. Quantitative systematic reviews generally adopt the internationally accepted principles of Picos. In this principle, p (population) refers to the study subject, i.e., the group in which the research subjects are enrolled; I (intervention) refers to interventions, i.e., interventions to test the effectiveness; C (comparison) refers to the control measure, i.e., the control group or another intervention; O (outcome) refers to an outcome measure, i.e., the specific measure to determine the effectiveness of an intervention; S (study design) refers to the type of study, i.e., the type of study of the original literature, which generally involves randomized controlled trials (RCTs), non RCTs, and cohort studies. Among them, RCTs are considered to be the type with the highest study quality^21^.

Inclusion criteria: (1) subjects: schooled students, excluding students in special education, regardless of gender, race, or nationality; (2) interventions: intervention model for the use of exergames in PE teaching; (3) control measures: traditional teaching methods synchronized with the experimental group; (4) outcome measures: the learning outcomes of the participants, such as tests of exercise capacity, physical activity, and motivation, were used as outcome measures; (5) study type: RCTs or quasi-experiment.

Exclusion criteria: (1) repeatedly published literature; (2) studies without specific experimental test scores, unable to effectively extract important data, and relevant data not obtained by contacting the researchers; (3) conference, degree, and reviews; (4) non-Chinese and English literature.

### Literature screening

Two researchers independently screened the literature and extracted data. Due to the large number of literature in the initial screening, only the titles and abstracts were read, and those that did not meet the inclusion criteria of Picos were removed. Secondly, for potentially eligible studies, those that failed to meet research requirements and designs were excluded after carefully reading the full text. Finally, the literature screening results were checked by two investigators, and a third person was consulted in case of disagreement.

### Risk of bias assessment

After literature screening, those that met the inclusion criteria were assessed for risk of bias to guarantee the internal authenticity of the meta-analytic results. The quality of the screened literature was assessed using the Cochrane risk of bias assessment tool^22^, which contains seven indicators: random assignment method, concealment of allocation scheme, implementation bias, measurement bias, follow-up bias, reporting bias, and other biases. Based on these seven indicators, a judgment of "yes" (low risk), "no" (high risk), or "unclear" (lack of relevant information or uncertain circumstances of bias) was made for each study. With more 'low-risk' entries, there is less potential for various types of bias, resulting in higher study quality ratings. This process was conducted independently by two investigators, and a third person was consulted in case of disagreement.

### Statistical analysis

Effect size pooling, heterogeneity testing, and subgroup analysis were performed on the outcome indicators using Revman 5.4 software. The results of this study were all continuous variables, and effect sizes were calculated as standardized mean difference (SMD) at a confidence level of 95% CI to assess the effect of exergames on student PE learning. P < 0.05 was considered statistically significant. Following the classification criteria of Cohen^23^, the effect size SMD < 0.2 indicates a small effect; 0.2 ≤ SMD < 0.5 represents a medium effect; 0.5 ≤ SMD < 0.8 indicates a medium to moderate effect; SMD ≥ 0.8 indicates a large effect.

The selection statistic I^2^ represents heterogeneity among included studies, reflecting the degree of overlap in the confidence intervals. It does not depend on the true effect size and distribution but rather reflects the magnitude of differences in relative proportions^24^. I^2^ = 0 suggests no heterogeneity across studies; 0 < I^2^ < 20% indicates a low heterogeneity between studies; 20% < I^2^ < 50% indicates a moderate heterogeneity between studies; I^2^ > 50% indicates a high heterogeneity among studies. When I^2^ > 50%, a random effect model was selected, and the subgroup analysis and sensitivity analysis were performed^25^.

### Data extraction and eigenvalue coding

After literature screening, the contents of relevant information were extracted, including: (1) basic information, such as author and publication year; (2) study subjects, such as total number and education level; (3) interventions, such as the number of experimental versus control subjects and experimental period; (4) the measurements, including the total number, mean, and standard deviation of each group of the experimental and control students. For subsequent analyses, the eigenvalues of the included studies were coded. According to the research topic of this study, the independent variable was exergames, the dependent variable was PE learning efficiency, and the dependent variables were coded as cognitive class A (academic achievement, exercise capacity, physical quality, theoretical knowledge, practical skills, etc.) and non-cognitive class B (learning motivation, learning interest, learning attitude, mental health, etc.). In addition, stage, class size, and experimental period were included as moderating variables. The specific coding rules are as follows: (1) classifying school stages into the child, elementary school, and secondary school; (2) categorizing the class size into small class size (0–30 participants), medium class size (31–50 participants), and large class size (more than 50 participants) according to the student number. (3) experimental cycles were divided into 0–1 month, 1–2 months, and ≥ 3 months according to the experiment duration.

## Results

### Search results

**Figure 1:**
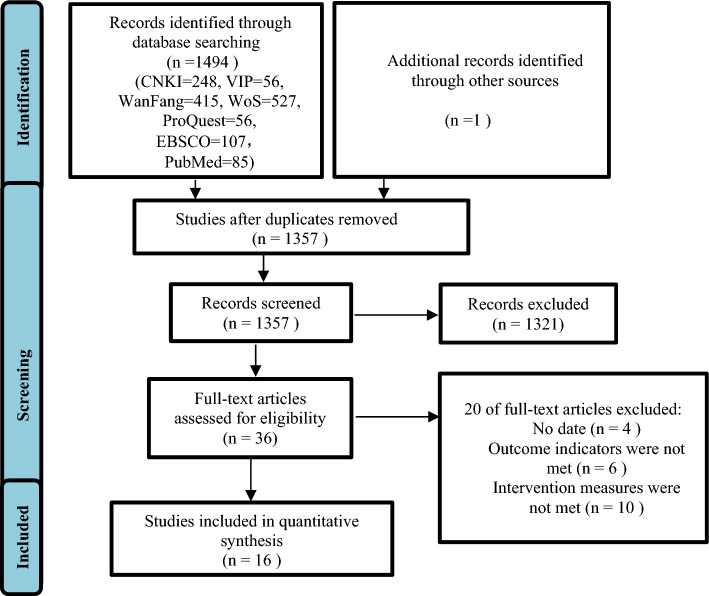
Flowchart for literature screening.

Through an initial search of seven electronic databases, 1494 relevant studies were obtained and imported into the citation manager EndNote X9 software. A total of 137 duplicate studies were identified and eliminated, and 1357 records were further screened according to their titles and abstracts. After a brief reading, 1321 studies unrelated to the research topic or not meeting the inclusion criteria were excluded, and the full text of the remaining 36 studies was downloaded. After further reading of the article, 20 studies with incomplete data, non-RCTs, and interventions and outcome indicators that did not meet the standards were excluded, and 16 studies that met the standards were included for the meta-analysis. The literature screening process and results are shown in Fig. 1.

### Study characteristics

The characteristics of the 16 included studies are shown in Table 1. Among the 16 included studies, all were published between 2012 and 2023, with a total of 2962 subjects, including 1500 in the experimental group and 1462 in the control group. The subjects include students in early childhood, elementary school, and secondary school, with a class size of less than 50 students. The experimental period ranged from a minimum of 3 acute interventions to a maximum intervention duration of 9 months. The learning effect includes indicators of cognitive ability (e.g., motor ability and physical activity) and non-cognitive ability (e.g., motivation).

**Table 1:** Characteristics of published studies included in this meta-analysis.

Study	Number of students (E/C)	Stage	Class size	Experimental period	Learning effect
Andrade (2019)^26^	66:72	Elementary	Small	0–1 month	B
Liu ZM (2022)^27^	22:24	Child	Small	0–1 month	A
Ye, SY (2018)^28^	135:115	Elementary	Small	≥ 3 months	A
Sheehan, DP (2013)^29^	21:21	Elementary	Small	1–2 months	A
Gao Zan (2017)^30^	85:79	Elementary	Small	≥ 3 months	A
Gao Zan (2019)^31^	20:36	Child	Middle	1–2 months	A
Lwin, Mo (2012)^32^	557:555	Elementary	Middle	1–2 months	A、B
Sun Haichun (2012)^33^	46:42	Elementary	Small	≥ 3 months	B
Quintas, A (2020)^34^	226:191	Elementary	Small	≥ 3 months	A
Jose Serrano (2021)^35^	17:19	Secondary	Small	0–1 month	A
Han Chen (2017)^36^	34:28	Elementary	Small	1–2 months	A
Victor Jaoier (2022)^37^	133:142	Secondary	Middle	0–1 month	B
Hsiao (2016)^38^	52:53	Child	Small	≥ 3 months	A
Ye Qiang (2017)^39^	18:19	Elementary	Small	1–2 months	A
Xiong SY 2019^40^	30:30	Child	Small	1–2 months	A
Klovelonis 2023^41^	38:36	Elementary	Small	0–1 month	A

*E/C* experimental group/control group, *A/B* cognitive ability/non-cognitive ability.

### Study quality

**Figure 2:**
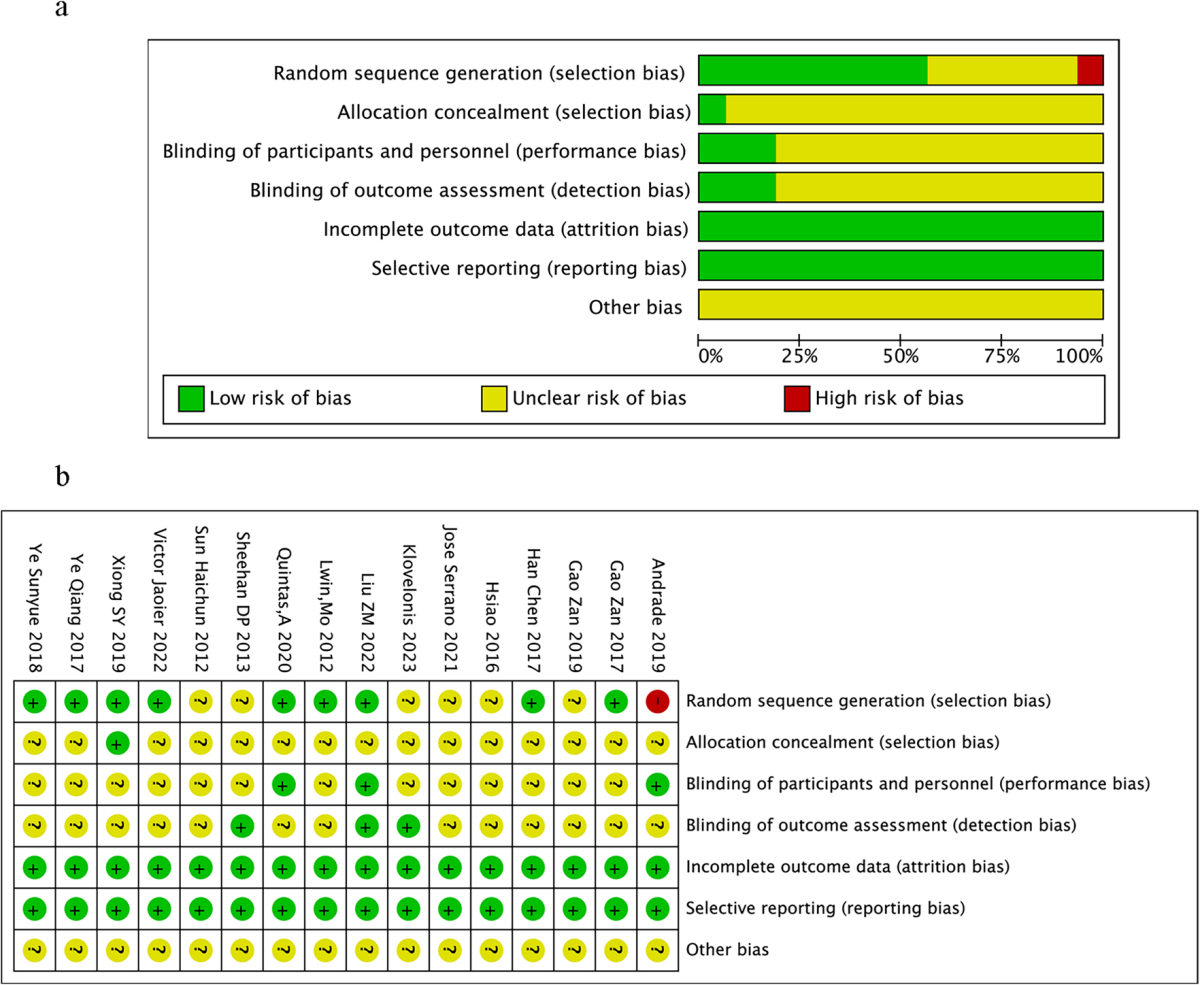
(**a**) Risk of bias graph: review authors’ judgements about each risk of bias item presented as percentages across all included studies. (**b**) Risk of bias summary: review authors’ judgements about each risk of bias item for each included study.

The methodological quality of each study is summarized on the left side of Fig. 2. Among 16 included studies, nine described random allocation concealment, while the others did not mention it or described it in little detail. Two included studies involved allocation concealment, while the remaining literature did not introduce the allocation or concealment plan. In addition, only three studies have implemented blind methods for participants and outcome evaluations. Almost every study truthfully reported outcome data and research results. The reasons and treatment methods for those who withdrew during the experiment were also explained. Therefore, follow-up bias and reporting bias were relatively low.

### Publication bias

**Figure 3:**
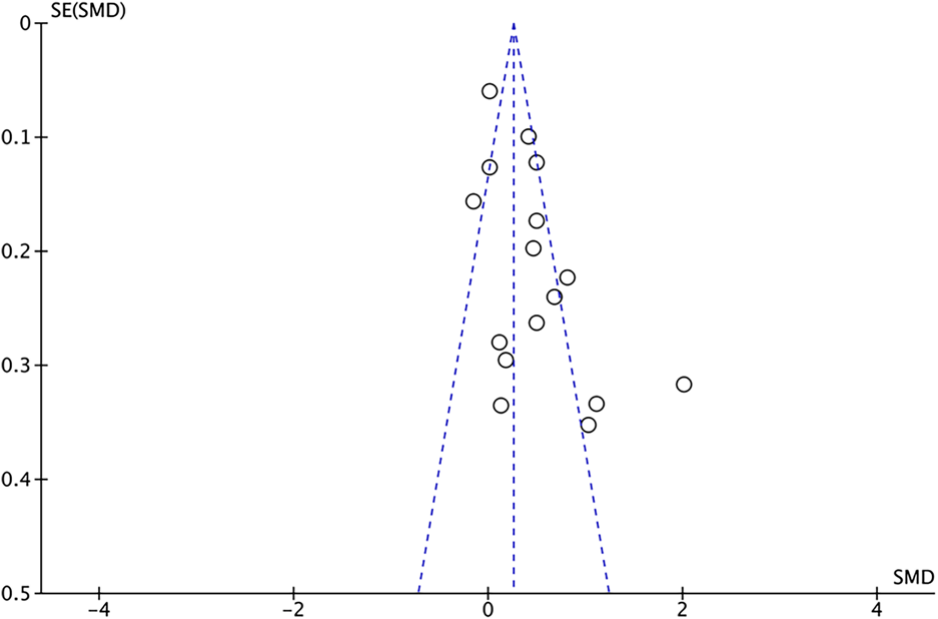
Funnel plot for publication bias testing.

As sample sizes and research methods vary among different studies, and the included studies do not systematically show the effects of interventions, it is necessary to evaluate publication bias. When the number of included studies in the meta-analysis is greater than or equal to 10, the funnel plot method can be adopted for publication bias assessment. From the distribution of the 16 studies in the funnel plot (Fig. 3), the effect sizes of samples are distributed on both sides of the mean effect value and basically in the upper middle panel. The publication bias is small, and the detected results can be used for subsequent analysis.

To accurately explore the impact of exergames on student PE learning outcomes, the heterogeneity test was performed to select an appropriate effect model. The commonly used methods for the heterogeneity test are the Q-test and I^2^ test, and the test model is selected based on the significance of statistical heterogeneity. In the case of significance (I^2^ ≥ 50%, P < 0.05), a random effect model can be used; in the case of non-significance (I^2^ < 50% , P ≥ 0.05), a fixed effect model needs to be adopted^42^. In this study, the heterogeneity test results are Q = 100.98, P < 0.0001, and I^2^ = 83%, indicating a high degree of heterogeneity among the included studies. Therefore, a random effect model is selected for evaluation.

### The overall impact of exergames on student PE learning outcomes

#### The overall effect size of exergames

The effect size is an indicator to assess the strength of experimental effects and the degree of correlation. Because the means of the included studies were different, SMD was used as the effect size for the analysis. The results obtained from the random effect model revealed that SMD = 0.45 (P < 0.00001), indicating a moderate positive effect of exergames on student PE learning (Table 2).

**Table 2:** The overall effect of exergames on student PE learning.

Model	Effect size	SMD	95% CI		Two-tailed test		Heterogeneity test			
			Lower limit	Upper limit	Z	P	Q	df	P	I^2^
Fixed model	18	0.21	0.15	0.27	6.73	< 0.00001	100.98	17	< 0.00001	83%
Random model	18	0.45	0.27	0.63	4.95	< 0.00001				

### Effects of exergames on cognitive and non-cognitive dimensions in students.

Table 3 shows that exergames have a moderate positive effect on student learning outcomes in the cognitive dimension (SMD = 0.47, P < 0.0001). In the non-cognitive dimension, exergames also have a moderate positive effect on student learning outcomes (SMD = 0.44, P = 0.01). The level of statistical significance is achieved in both dimensions.

**Table 3:** Effects of exergames on learning in cognitive and non-cognitive dimensions.

Learning effect	Effect size	SMD	95% CI		Two-tailed test	
			Lower limit	Upper limit	Z	P
Cognitive dimension	14	0.47	0.23	0.71	3.90	< 0.0001
Non-cognitive dimension	4	0.44	0.10	0.78	2.53	0.01

### Effects of different moderating variables of exergames on student PE learning outcomes

To explore the effects of different moderating variables on student PE learning, statistical analyses were conducted with different classes, different class sizes, and different teaching cycles.

### Effects of exergames on student PE learning with different stages

Modern developmental psychology suggests that the cognitive development of children and adolescents should be divided into stages according to their growth process. Students need to go through different stages of cognitive development, from concrete and superficial to abstract, as well as from perception and induction to reasoning^43^. Considering the growth and development patterns of students, there is an asynchronous relationship between their cognitive and non-cognitive abilities. Due to insufficient experimental investigations on the development of non-cognitive abilities, only differences in the impact of exergames on the cognitive abilities of students in the PE class can be presented when analyzing different groups of students. Therefore, this study only focuses on cognitive ability and discusses the effects of somatosensory games in different age groups. This study categorized the academic stages of the included studies into child stage, elementary school, and secondary school. The data in Table 4 show that the effect size SMD = 0.44(P = 0.0008 < 0.05) for young children, SMD = 0.40 (P = 0.0002 < 0.05) for elementary school, and SMD = 0.14(P = 0.67 > 0.05) for secondary school, indicating that exergames have a moderate positive effect on PE learning among students. Since the effect value for the secondary school fails to reach a statistically significant level (P > 0.05), it cannot be demonstrated that exergames have a significant effect on the PE learning of students in secondary school.

**Table 4:** Effects of exergames on student PE learning with different moderating variables.

Coding object	Type	Effect size	SMD	95% CI		Two-tailed test	
				Lower limit	Upper limit	Z	P
Stage	Child stage	5	0.44	0.18	0.69	3.37	0.0008
	Elementary school	9	0.40	0.15	0.65	3.14	0.002
	Secondary school	1	0.14	–0.15	0.80	0.42	0.67
Class size	Small class size	13	0.51	0.26	0.77	3.93	< 0.0001
	Medium class size	5	0.24	0.02	0.46	2.15	0.03
Experimental cycle	0–1 month	5	0.47	0.31	0.64	5.54	< 0.00001
	1–2 months	8	0.62	0.30	0.94	3.77	0.0002
	≥ 3 months	5	0.29	-0.01	0.59	1.92	0.05

### Effects of exergames on student PE learning with different class sizes

In order to explore the effect of exergames on student PE learning with different class sizes, two class sizes (small and medium) were defined for the analysis. Table 4 shows that the effect size of the small class size is the highest, SMD = 0.51 (P < 0.0001), achieving a moderate to high positive effect. The medium class size shows an effect size of 5, SMD = 0.24 (P = 0.03 < 0.05), achieving a moderate positive effect. These results indicate that exergames positively affect student PE learning with different class sizes.

### Effects of exergames on student PE learning with different experimental cycles

This study also explored the effects of exergames on student PE learning with different experimental cycles, and the included studies were divided into 0–1 month, 1–2 months, and ≥ 3 months according to the experimental cycle. Table 4 shows that the highest effect size is achieved at 1–2 months with SMD = 0.62 (P = 0.0002 < 0.05), indicating a moderate to high degree of positive effect. The size effect of 0–1 month is 5, SMD = 0.47 (P < 0.00001), indicating a moderate positive effect. As the effect size for ≥ 3 months fails to reach the statistical significance level (P = 0.05), it has no significant effect on student PE learning. Based on the above analysis, the effect of exergames is related to the experimental cycle, but a longer duration does not represent a better effect.

## Discussion

The statistical analysis demonstrates that exergames can promote student PE learning, with positive effects in both cognitive and non-cognitive dimensions. In addition, the effect of exergames on student PE learning outcomes varies with different moderating variables.

The meta-analysis results show that the exergames have a moderate positive effect on student PE learning (SMD = 0.45, ranging from 0.2 to 0.5), indicating that the adoption of exergames can facilitate student PE learning with statistical significance. The reasons may include the following aspects: (1) Exergames are characterized by entertainment, flexibility, and competition. By learning in the game, students can improve their participation and interest in learning, thus motivating them to study actively. In addition, students gain a sense of accomplishment in completing the game tasks, further improving their self-confidence in learning^44^. (2) Exergames can provide a relaxing and enjoyable learning environment. Students can perform multiple and repeated simulation exercises in this environment, thus creating a stronger sense of actual participation and experience, fully feeling the pleasure and charm of sports. In addition, the learning environment created by games allows students to establish a general perception of learning from technical movements^45^. (3) Children and adolescents begin interacting with technical devices at an early age and are immensely obsessed with virtual games^46^. The incorporation of exergames in the PE programs precisely matches the preferences of students. As a result, students are transformed from passive, receptive learning habits to spontaneous, active learning, ultimately achieving a high quality of learning. In conclusion, exergames are in line with the modern concept of education, aiming at the development of students and the improvement of health quality and social adaptability^47^.

In terms of specific learning effects, exergames significantly promote the cognitive and non-cognitive abilities of students. Researchers argued that intrinsic motivation arises from the natural interest of humans in activities that offer novelty, surprise, curiosity, or challenge^48^. When engaging in such activities, the brain can feel dynamic pleasure, excitement, or gratification, prompting attention to be focused on the process (e.g., learning about unknown knowledge, mastering new skills, etc.) rather than on external signals of reward or punishment^49^. Just as playing is a child's innate talent^50^, Exergames provide a platform for students to fully express themselves. Through the medium of games, students can boldly present themselves, and strong intrinsic motivation drives them to be more actively involved in PE class. Rewards for success in the game encourage students to feel pride and satisfaction and gradually enhance their self-confidence and self-identity, leading to a positive learning attitude, increased frequency of practice, and continuous improvement of skills during the course.

Furthermore, the moderating variables were divided into three categories according to the needs of the study: stage, class size, and experimental cycle.

In terms of stage, exergames positively affect students at all grade levels, with the greatest effect on young children, followed by elementary school students, with the least effect on secondary school students. The reason for this is that playing games is an innate nature of children. Exergames can provide children with positive emotional experiences and enjoyment, thus increasing their intrinsic motivation to exercise. Children who use exergames are more motivated and are more engaged in exercise than in traditional sports^51^. Based on the literature on immersion study in exergames, once children are immersed in exergames, their perceptions of the game intensity might change, resulting in higher and longer motivation to play and exercise^52^. The thinking of elementary school students is based on image thinking, and the games can help them understand abstract knowledge through concrete things, thus promoting learning effects^53^. In contrast, secondary school students have received regular PE for many years. They have been accustomed to strict normative requirements, following common standards under uniform instructions, and learning technical specifications of exercise summarized by humans. If they repeat the didactic approach of the game, they will not be able to achieve the original effect. Therefore, PE teachers should select appropriate exergames based on the mental development and sports learning environment of students.

In terms of class size, the best effect of exergames is achieved in the small class. This result is consistent with the general consensus on the relationship between the class size and the learning performance^54^. Gametic teaching is more advantageous in classes with relatively small sizes, fulfilling requirements for deep participation, positive interaction, and timely feedback^55^. Typical exergames rely on corresponding somatosensory devices. Due to the high equipment cost, schools may only have a single device shared by all students, and smaller class sizes may enable students to efficiently participate in activities on a somatosensory platform. In contrast, a large class size may affect the effectiveness of exergame activities. Thus, implementing exergames in classes with small sizes is effective.

In terms of experimental cycles, different experimental cycles affected student PE learning to varying degrees, with 1–2 months having the greatest effect, 0–1 month the second, and ≥ 3 months experimental cycle having the least effect. Long-term game interventions are less effective than short-term play interventions, consistent with the previous study^56^. This finding suggests that learners may be more motivated to learn or participate in intensive short-term activities than in extended education settings^57^. In the early stage of learning, students have a great interest in exergames, thus greatly promoting their enthusiasm for learning. The 0–1 month experimental cycle is in the learning phase of using somatosensory technologies, failing to bring students a positive game experience. It is accompanied by a higher level of familiarity with exergames, less enthusiasm and freshness of learning, and lower academic engagement. When the novelty of a playful learning experience is transformed into an ordinary experience, the positive effects of exergames disappear^58^. Therefore, it is important for PE teachers to choose diverse exergames to enhance the durability of their effects.

## Conclusion

In summary, exergames can effectively promote student PE learning and positively affect their cognitive and non-cognitive abilities. To achieve a better intervention effect, PE teachers need to select the corresponding games according to the cognitive level of students in different age groups, preferably in small classes, and limit the implementation cycle to 1–2 months. It is recommended that PE teachers make full use of big data, artificial intelligence, and other information technologies to combine students' interest in video games with PE curriculum using exergames as a medium. In the process of PE teaching, a good gametic learning environment needs to be created to fully utilize exergames to promote the high-quality development of school sports (Supplementary Information).

### Supplementary Information

Supplementary Information.

## Data Availability

The datasets used and/or analysed during the current study available from the corresponding author on reasonable request.
