# Supplementary material for: Effects of exergames on student physical education learning in the context of the artificial intelligence era: a meta-analysis
Source: Sci Rep. 2024 Mar 26;14:7115. doi: 10.1038/s41598-024-57357-8 (PMC10965939; doi:10.1038/s41598-024-57357-8)

**Table and Source Data**

**Source of Table 1:**

| Study | Number of  students  (E/C) | Stage | Class size | Experimental period | Learning effect |
| --- | --- | --- | --- | --- | --- |
| Andrade (2019) | 66:72 | elementary | small | 0-1 month | B |
| Liu ZM (2022) | 22:24 | child | small | 0-1 month | A |
| Ye, SY (2018) | 135:115 | elementary | small | ≥3 months | A |
| Sheehan, DP (2013) | 21:21 | elementary | small | 1-2 months | A |
| Gao Zan (2017) | 85:79 | elementary | small | ≥3 months | A |
| Gao Zan (2019) | 20:36 | child | middle | 1-2 months | A |
| Lwin, Mo (2012) | 557:555 | elementary | middle | 1-2 months | A、B |
| Sun Haichun (2012) | 46:42 | elementary | small | ≥3 months | B |
| Quintas, A (2020) | 226:191 | elementary | small | ≥3 months | A |
| Jose Serrano (2021) | 17:19 | secondary | small | 0-1 month | A |
| Han Chen (2017) | 34:28 | elementary | small | 1-2 months | A |
| Victor Jaoier (2022) | 133:142 | secondary | middle | 0-1 month | B |
| Hsiao (2016) | 52:53 | child | small | ≥3 months | A |
| Ye Qiang (2017) | 18:19 | elementary | small | 1-2 months | A |
| Xiong SY (2019) | 30:30 | child | small | 1-2 months | A |
| Klovelonis (2023) | 38:36 | elementary | small | 0-1 month | A |

**Source of Table 2：**

**(1)Random model**


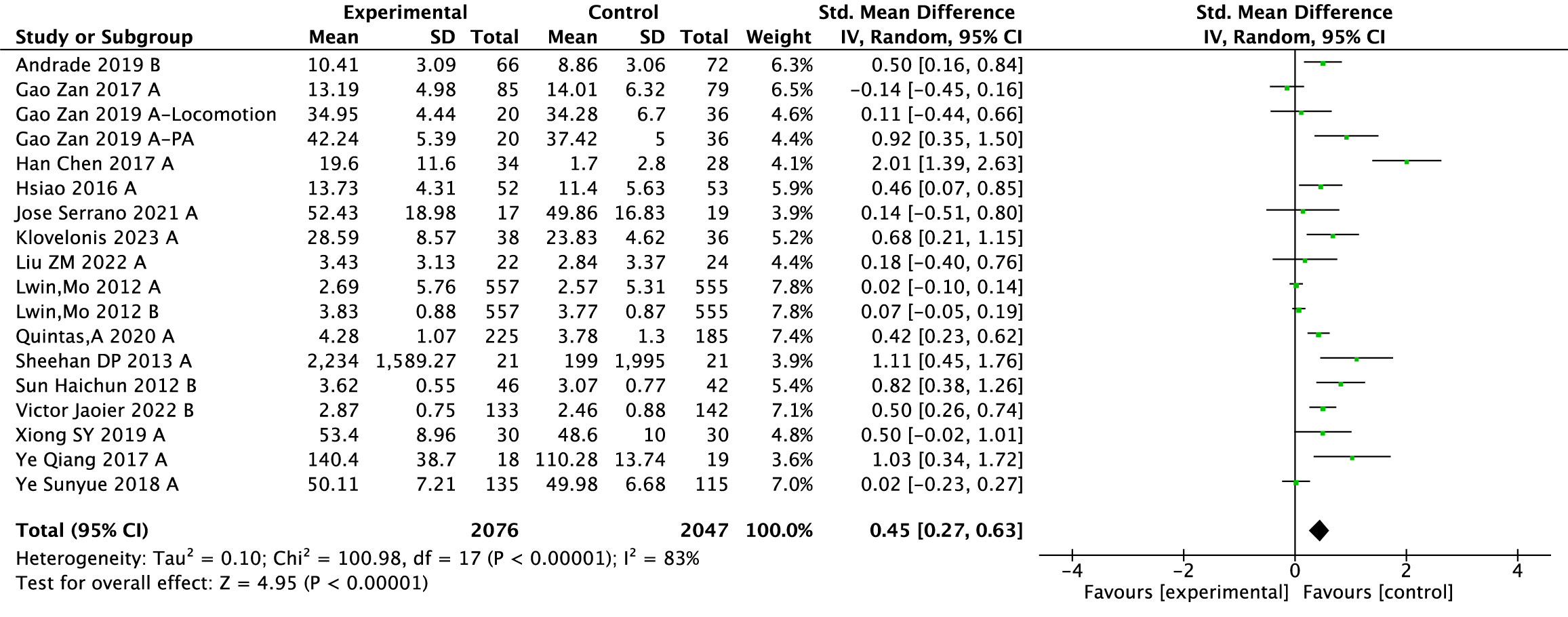


**(2)Fixed model**


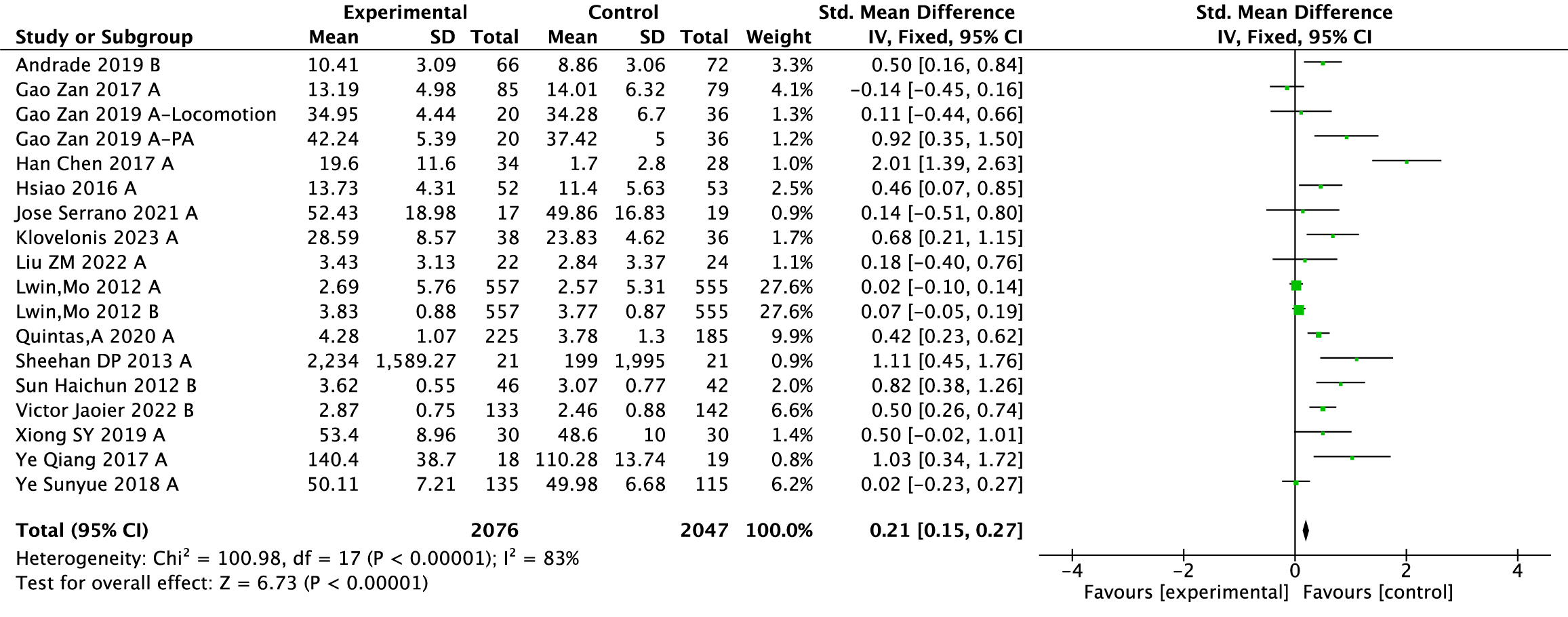


**Source of Table 3--Cognitive and Non-cogonitive dimension：**


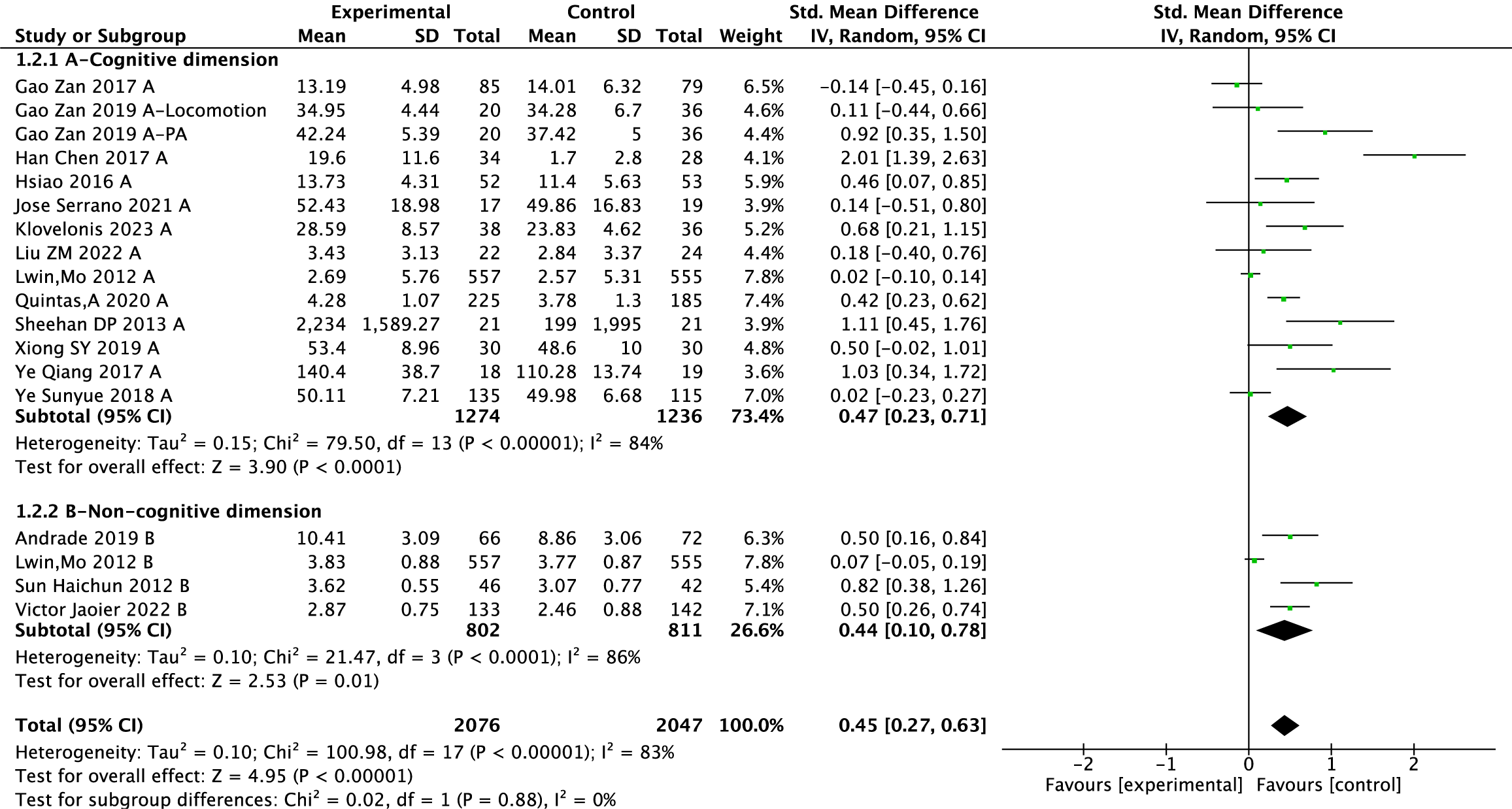


**Source of Table 4：**

**（1）Period**


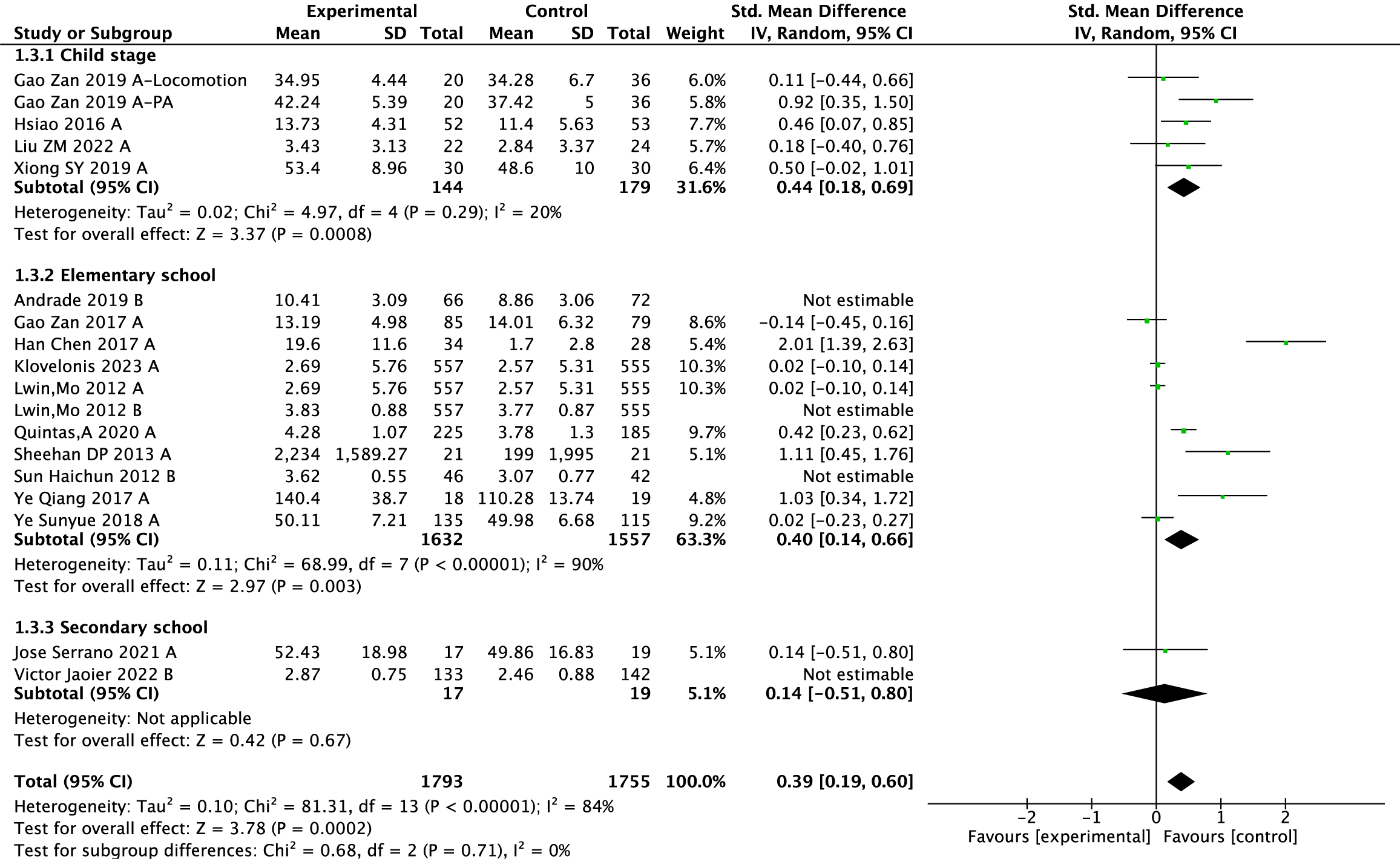


**（2）Class Size**


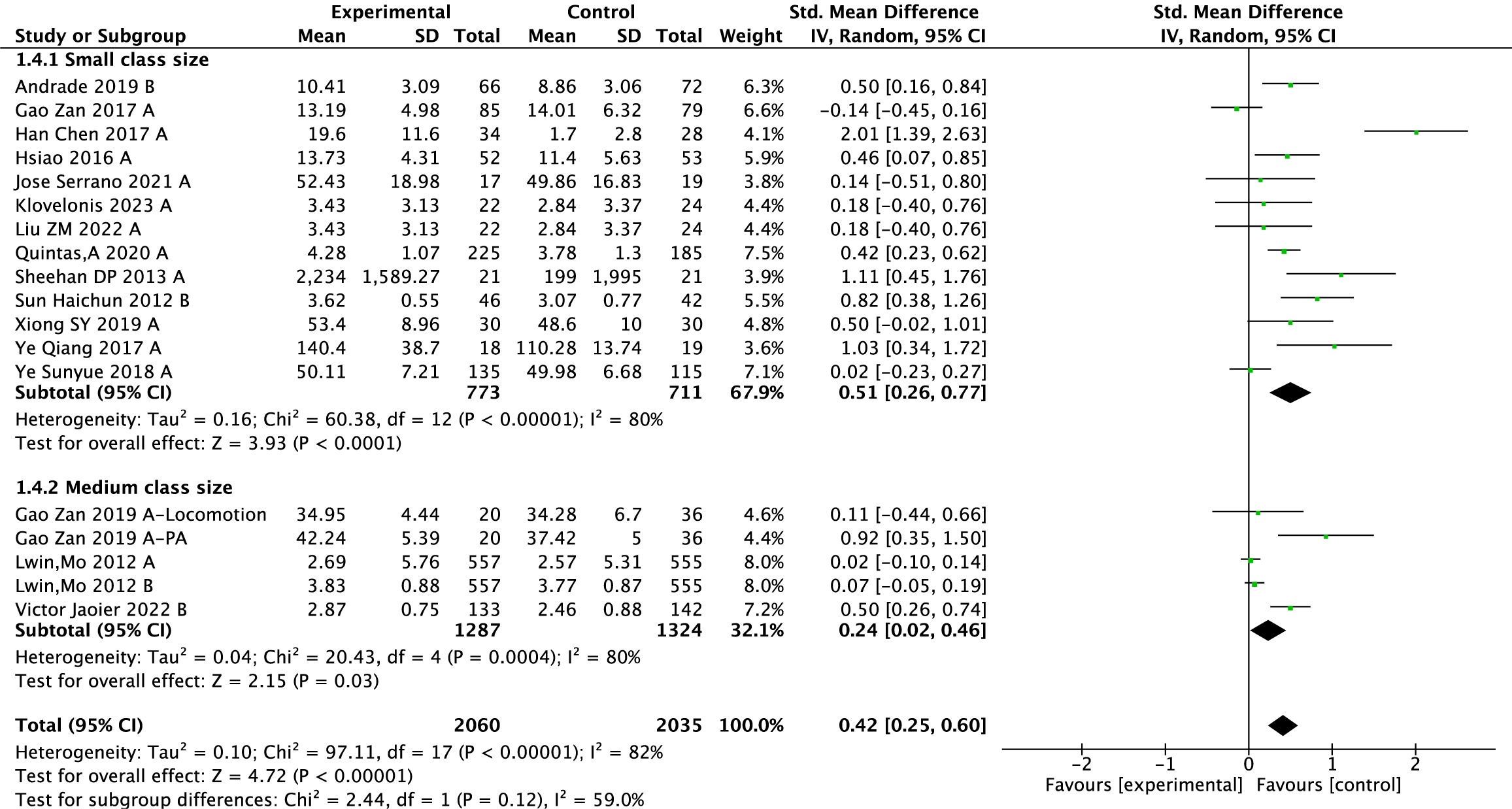


**（3）Experimental cycle**


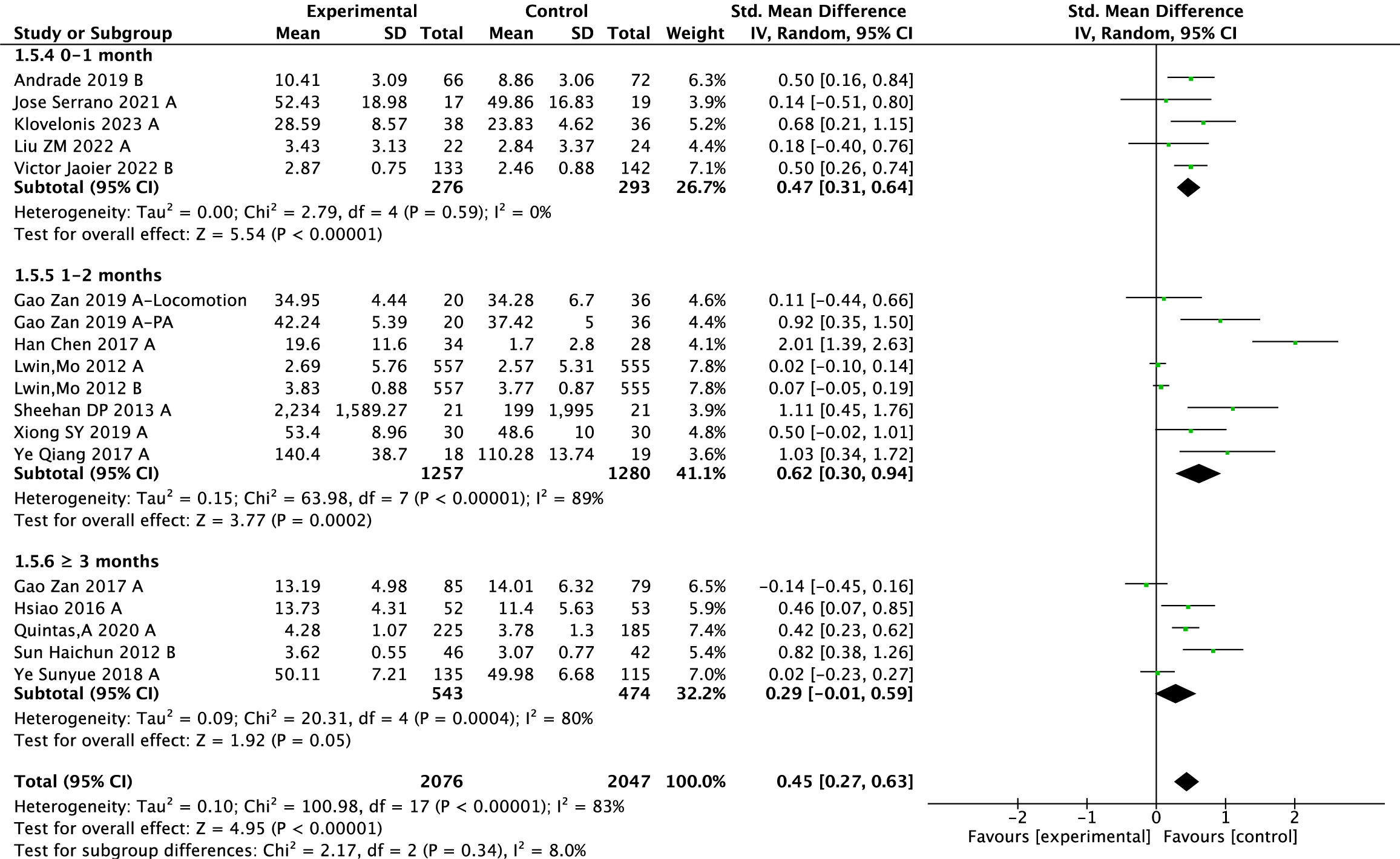

Supplement: Supplementary file 1 — Supplementary Information. [file 41598_2024_57357_MOESM1_ESM.docx]
